# Research Progress of Hair Cell Protection Mechanism

**DOI:** 10.1155/2020/8850447

**Published:** 2020-10-09

**Authors:** Yurong Mu, Hongguo Su, Fan Wu, Jianming Yang, Dan Li

**Affiliations:** ^1^Department of Otorhinolaryngology, Union Hospital, Tongji Medical College, Huazhong University of Science and Technology, Wuhan 430022, China; ^2^Department of Otorhinolaryngology, Head and Neck Surgery, Zhongnan Hospital of Wuhan University, Wuhan 430071, China; ^3^Otorhinolaryngology Department, Sun Yat-sen Memorial Hospital, Sun Yat-sen University, 107 West Yan Jiang Road, Guangzhou 510120, China; ^4^Department of Otorhinolaryngology, The Second Affiliated Hospital of Anhui Medical University, Hefei 230601, China

## Abstract

How to prevent and treat hearing-related diseases through the protection of hair cells (HCs) is the focus in the field of hearing in recent years. Hearing loss caused by dysfunction or loss of HCs is the main cause of hearing diseases. Therefore, clarifying the related mechanisms of HC development, apoptosis, protection, and regeneration is the main goal of current hearing research. This review introduces the latest research on mechanism of HC protection and regeneration.

## 1. Introduction

Hearing loss has become one of the common health problems in the world, and more and more people suffer from deafness. In recent years, with the exploration and understanding of the mechanism of deafness, many researchers hope to improve or restore hearing through promoting HC survival or regeneration. We will introduce the research progress and new findings of the mechanism of protecting and regenerating in HCs in this manuscript.

## 2. The Development of HCs

The inner ear of the mammal originates from the ectoderm of the embryo, and the ectoderm is locally thickened to form the auditory placode, and then the auditory placode is recessed to form an otic cup. When the otic cup closes off, a sac-like otic vesicle is formed [1, 2]. At E10.5-11, the cochlea duct is derived from the ventral side of the otic vesicle, and the bottom layer of the cochlea forms the proneurosensory domain. At E14.5, the differentiation of sensory precursor cells was initially from the central domain in the basal of the cochlea, which progressively extends toward the apex and basal, and finally differentiates into inner ear cells such as HCs and supporting cells (SCs) [3]. In this process, the differentiation of sensory precursor cells into HCs and SCs mainly depends on the expression of the transcription factor Math1 (also known as Atoh1, [4]). The development of the inner and outer HCs progressed gradually from the basal turn to the apex turn, and the inner HCs (IHCs) develop earlier than the outer HCs (OHCs) [5–8].

HCs are located on the organ of Corti, which includes a row of IHCs and three rows of OHCs and SCs. The OHCs mechanically amplify and detect low-level sound, thereby enhancing the responsiveness of the sensory epithelium to different sound frequencies. The IHCs transmit the sound stimulation to the nerve; thereby, the sound stimulation is transformed into nerve excitement electrical signals, which is then transmitted along the auditory nerve to the auditory center to generate hearing [9]. SCs provide structural and nutritional support for the long-term survival of HCs.

## 3. The Apoptosis of HCs

HC apoptosis is a process that occurs programmatically under the control of genes and involves multiple triggering factors and signaling pathways. As we all know, ototoxic drugs, noise, aging, and other factors can cause apoptosis of HCs, and the main mechanisms of apoptosis can be roughly divided into the following two.

### 3.1. Exogenous Pathway of HCs Apoptosis

Exogenous pathways can be triggered by a variety of factors, such as cell surface death receptor (FasL), which can cause the recruitment of specific adaptor proteins and the activation of caspases-8 and caspases-10. Other exogenous receptors include tumor necrosis factor alpha (TNF-*α*), tumor necrosis factor-related apoptosis-inducing ligand (TRAIL), and death receptor (DR), each of which can affect specific intracellular medium and eventually activate caspase (a protease family that plays an important role in programmed cell death and inflammation). In addition, TNF-*α* can also activate JNK, inhibit the antiapoptotic factor Bcl-2 through phosphorylation, and eventually cause apoptosis [10].

### 3.2. Endogenous Pathway of HC Apoptosis

The endogenous apoptosis pathway of HCs can be activated by intracellular stressors, such as reactive oxygen species (ROS). Previous studies have reported that HC loss caused by ototoxic drugs is usually associated with ROS accumulation, which can induce mitochondrial depolarization and trigger apoptosis [11]. Mitochondria are the main place for cells to produce ROS. Normally, the production level of ROS is low, which plays an important role in signal transduction, immune response, and gene expression regulation. But when the accumulation of reactive oxygen species is excessive, it will have a toxic effect on the cells and eventually lead to apoptosis [12]. The appropriate level of reactive oxygen species can promote autophagy and renew damaged cell components, thereby maintaining the stability of the intracellular environment. Autophagy is a physiologically conservative multistep process for recycling endogenous or exogenous cytoplasmic materials, such as misfolded proteins, lipids, organelles (mitochondria and ribosomes), cellular components, and peroxide enzyme and virus or bacteria, and finally degraded after fusion with lysosome [13]. Previous studies have reported that induced autophagy can inhibit the accumulation of ROS after aminoglycoside damage, thereby playing a role in promoting HC survival [14]. In addition, endogenous apoptosis pathways can be activated not only by intracellular stressors (such as ROS), but also by activated exogenous pathways, which shows that the two pathways are not completely independent.

## 4. The Regeneration of HCs

Most inner ear HCs of mammals are formed in the embryonic stage. After birth, due to congenital infections, noise, and improper use of ototoxic drugs such as aminoglycoside antibiotics, the HCs are irreversibly damaged and eventually lead to permanent hearing impairment [15]. Unlike mammals, the inner ear HCs of vertebrates such as fish and birds can be restored to normal levels after HC regeneration, mainly through the following two ways: the mitotic regeneration, the SCs proliferate and divide first and then a part of the SCs differentiate into HCs. In the direct transdifferentiation, the SCs directly differentiate into HCs without undergoing proliferation and division [16].

Adult mammalian cannot spontaneously generate new HCs, but in recent years, studies have found that HCs, SCs, and pluripotent stem cells near the damaged area of the inner ear may be the source of HC regeneration under certain conditions. HC regeneration involves coordinated regulation of multiple factors and signaling pathways. In SC transdifferentiation-related research, it was found that the transcription factor Hes/Hey in the Notch signaling pathway can affect cell fate during development by combining the *Atoh1* promoter region. In addition, the regulation of multiple cell fate-related genes such as *p27*, *Atoh1*, and *Pou4f3* can enable the functional regeneration of HCs in adult animals. The role of epigenetics in HC regeneration has also been proven. For example, histone demethylase LSD1 affects the expression of genes related to HC development and transdifferentiation by regulating the level of H3K4 methylation modification [17]. miRNAs have been shown to participate in the regulation of the expression of many important genes during cochlea development, thereby affecting the processes of cochlea cell proliferation, migration, development, and apoptosis [18]. For example, miR-183 and miR-210 were found to play an important role in the transdifferentiation of SCs to HCs [19, 20]. In addition, the control of the number of regenerated HCs, the reconstruction of cell structure, the effective arrangement of cilia, the fine docking of nerve synapses, etc. are all important basis for evaluating the success of HC regeneration [21]. Through continuous exploration of the mechanism of sensory HC regeneration, it will help to solve the problem of mammalian sensory HC regeneration in the future.

## 5. HC Regeneration-Related Signaling Pathways

During the development of cochlea HCs, a variety of signaling pathways and signaling molecules are involved in regulating their differentiation and development, including Notch, Wnt, BMP, FGF, Shh, and JNK [22]. Inhibition or activation of related signaling pathways can increase the number of transdifferentiated or proliferated HCs after damage [23].

### 5.1. The Role of Notch Signaling Pathway in HC Regeneration

Notch signaling molecules and their receptors are membrane integrins. Cells can directly receive signals from neighboring cells through the receptors on their surface and transmit them to the nucleus, thereby activating the expression of related transcription factors in the nucleus [24, 25]. The Notch signaling pathway plays a variety of roles in the development of the inner ear, from the formation of the ear in the embryo to the generation of SCs, HCs, and neurons [26]. Notch signaling can inhibit the differentiation of sensory precursor cells into HCs in adult rats, but after inner ear injury, the activation of Notch signaling pathway has the potential to promote HC regeneration [27–29].

### 5.2. The Role of Wnt Signaling Pathway in HC Regeneration

The Wnt signaling pathway is a highly conserved signaling pathway in the early stages of biological development and participates in regulating physiological processes such as embryonic development and differentiation, cell proliferation and growth, and cell polarity formation. The canonical Wnt/*β*-catenin signaling pathway regulates the specialization of the auditory placode and the differentiation of the otic vesicle in the early stages of inner ear development [30]. During the early development of the cochlea, the canonical Wnt signaling pathway is upregulated in cochlea precursor sensory cells, and the inhibition of Wnt/*β*-catenin signaling reduces HC formation in the differentiating organ of Corti [31]. In addition, the Wnt/PCP signaling pathway plays an important role in the arrangement of the stereocilia of HCs and the extension of the cochlea duct [32]. *Lgr5*^+^ cells in the cochlea of newborn mice have been shown to be an HC progenitor cell, which can regenerate HCs by direct differentiation or mitosis. Studies have found that inhibition of Notch can reactivate the inhibited Wnt signal, thereby promoting mitosis of Lgr5^+^ progenitor cells and generating new HCs [33].

### 5.3. The Role of BMP Signaling Pathway in HC Regeneration

Bone morphogenetic protein (BMP) belongs to the transforming growth factor beta (TGF-*β*) superfamily, and its ligands are expressed in the ear development of many animal species, including precursor sensory regions and sensory cells [34]. BMP signaling pathway plays an important role in gastrulation, mesoderm formation, and bone and nervous system development [35, 36]. Related studies of the inner ear have found that the BMP signaling pathway plays a regulatory role in the process of inner ear morphogenesis, formation and development of nerve fibers, HC development, etc. [37, 38]. When BMP signaling pathway is blocked, HCs and SCs decrease, and when exogenous BMP is added, HCs will increase [39].

### 5.4. The Role of FGF Signaling Pathway in HC Regeneration

Fibroblast growth factor (FGF) is an important intercellular signaling molecule. FGF plays a regulatory role in various physiological or pathological processes by binding to specific receptors on the cell membrane, such as embryonic development and organ formation, cell growth, tissue repair, tumorigenesis, and inflammation [40]. The FGF signaling pathway plays an important role in multiple stages of inner ear development, such as the formation of auditory placode and otic vesicle, the proliferation, and differentiation of sensory epithelial cells [41–43]. Studies have shown that inhibiting FGF can lead to the differentiation of precursor cells or SCs into HCs [44]. In the process of culturing inner ear sensory progenitor cells in vitro, ectopic activation of FGF receptor (FGFR) in mesenchyme is sufficient to increase sensory progenitor cell proliferation and cochlea length [45].

### 5.5. The Role of Shh Signaling Pathway in HC Regeneration

Shh is a class of Hedgehog (Hh) protein family, which regulates the gene expression of neighboring or distant cells in the form of extracellular secreted proteins and participates in regulating the development of multiple tissues and organs, affecting the occurrence of tumors and inducing tissue polarity [46, 47]. Shh is an important regulator of inner ear development and plays an important role in regulating inner ear morphogenesis, formation of spiral neurons, and differentiation of HCs [48]. Shh gene deletion can cause severe inner ear developmental disorders, such as malformation or loss of ventral structures (cochlea duct and saccule) and dorsal structure (semicircular canal, utricle, and endolymphatic ductus), and developmental disorders of Corti organs and ganglion nerve cells [49, 50]. However, there are still few reports on Shh signaling pathway in HC regeneration, so its regulatory mechanism remains to be elucidated.

## 6. HC Protection-Related Drug Applications

Currently known drugs that have protective effects on HCs mainly include JNK kinase inhibitors and antioxidant drugs. In mouse and guinea pig experiments, JNK kinase inhibitors can prevent hearing loss caused by noise exposure, ototoxic drug treatment, trauma, and other factors [51, 52]. The JNK kinase inhibitor AM-111 has now completed the phase III clinical trial. For severe sensorineural hearing loss, AM-111 has a good hearing protection effect [53]. Antioxidant drugs including N-acetylcysteine, ebselen (glutathione peroxidase mimic), D-methionine, vitamin E, and flunarizine all show hearing protection [54–56]. The hearing protection effect of antioxidant drugs may be related to the removal of free radicals and the synergistic effect on other antioxidant enzymes to maintain the integrity of the cell membrane and reduce the oxidative stress response of cells.

## 7. Application of Gene Therapy in HC Protection

Gene therapy refers to the treatment or prevention of diseases through the addition and expression of genes. These gene fragments can reconstruct or correct those missing or abnormal gene functions and can interfere with the pathogenic process. Adeno-associated virus (AAV) vector is a vector that transfers genes into cochlea cells. It has a highly efficient transduction effect and is safe and stable in terms of long-term expression. The vector constructed based on AAV1 can effectively transduce IHCs and spiral ganglion cells and introduce secreted proteins into the cochlea, thereby protecting inner ear sensory cells from drug-induced damage [57]. Although the AAV vector can transduce mouse cochlea IHCs, the OHCs are still difficult to transduce. There is also an AAV-ie viral vector that can efficiently transduce cochlea SCs and induce the SCs to transdifferentiation into HCs. With the continuous optimization of the AAV virus, Isgrig et al. have proved that AAV2.7m8 can efficiently infect cochlea IHCs and OHCs. In addition, AAV2.7m8 can also efficiently infect inner pillar cells and inner phalangeal cells [58]. These studies prove that the AAV virus as an excellent vector for inner ear gene therapy has a good application prospect.

## 8. Application of Exosomes in HC Protection

Exosomes are membrane-bound nanovesicles that contain a variety of biomolecules such as lipids, proteins, and nucleic acids. Exosomes are produced by cells through exocytosis and then taken up by target cells, which can transmit biological signals between local or distant cells [59]. Exosomes promote the interaction between HSP70 and TLR4 through intercellular communication, thereby activating non-cell-autonomous protective signaling in the inner ear and protecting HCs from aminoglycoside-induced damage [60]. Studies have found that exosome-associated AAV vectors have a higher efficiency of transducing cochlea and vestibular HCs than traditional AAV vectors [61].

## 9. Application of Biomaterials in HC Protection

Biomaterials are a type of artificial or natural materials that can be made alone or together with drugs for the treatment and replacement of tissues and organs and ultimately replace or repair human organs and tissues to achieve the remodeling of their physiological functions, without adversely affecting the body. The selection of biomaterials is particularly important for nerve regeneration, which requires a high biocompatibility with host tissues [62]. Researchers use the three-dimensional culture system and the regulation of various signaling pathways to cultivate pluripotent stem cells into inner ear organs containing functional HCs [63]. Compared with 2D, the 3D matrix gel culture system significantly promotes the growth of spiral ganglion explants and preserves the fine structure of spiral ganglion explants, so it can be used to simulate the three-dimensional structure of spiral ganglia under physiological conditions [64]. When therapeutic biomaterials enter the inner ear, they are restricted by the existence of biological structures and blood-brain barriers. Therefore, the construction of liposome nanoparticles or multifunctional nanoparticle-based drug delivery systems will help treat a variety of inner ear diseases [65].

## 10. Application of Stem Cell Therapy in Hearing Protection

Stem cell transplantation therapy uses stem cell pluripotency to restore or replace the function of spiral ganglion cells and HCs. Studies have found that bone marrow mesenchymal stem cells, embryonic stem cells (ESCs), adult inner ear stem cells, and neural stem cells can all become inner ear HC-like cells after inducing proliferation and differentiation [66]. When human pluripotent stem cell- (hPSC-) derived neurons are cocultured with rat HCs and cochlea nucleus neurons, hPSC-derived neurons are induced by inner ear HCs and cochlea nucleus neurons to form many new synapses [67]. It is known that Lgr5^+^ and Lgr6^+^ progenitor cells are a large number of progenitor cell groups present in the inner ear, both of which can be induced to generate HCs [68]. Although the research on inner ear stem cells has made great breakthroughs, there are still many problems waiting to be solved, such as the survival time of stem cells differentiated into cochlea sensory epithelial cells in the body and whether their physiological functions can function normally, how to deliver stem cells to the correct position, and how to avoid the body's immune rejection reaction.

## 11. Conclusion

The development and regeneration of HCs involve multiple factors and signaling pathways. At this stage, researchers have made many important discoveries about the protection and regeneration of HCs. With a more comprehensive understanding of the mechanism of HC regeneration, gene therapy and stem cell therapy will become important treatment options for the treatment of ear diseases in the future.

## Abbreviations

HCs: Hair cells
SCs: Supporting cells
IHCs: Inner HCs
OHCs: Outer HCs
FasL: Fas and Fas ligand
TNF-*α*: Tumor necrosis factor alpha
TRAIL: Tumor necrosis factor-related apoptosis-inducing ligand
DR: Death receptor
JNK: C-Jun N-terminal kinase
ROS: Reactive oxygen species
BMP: Bone morphogenetic protein
TGF-*β*: Transforming growth factor beta
FGF: Fibroblast growth factor
FGFR: FGF receptor
AAV: Adeno-associated virus
TLR4: Toll-like receptor 4
HSP70: Heat shock protein 70
ESCs: Embryonic stem cells
hPSCs: Human pluripotent stem cells.

## References

[1] Barald K. F., Kelley M. W. (2004). From placode to polarization: new tunes in inner ear development. *Development*.

[2] Fritzsch B., Pan N., Jahan I., Elliott K. L. (2015). Inner ear development: building a spiral ganglion and an organ of Corti out of unspecified ectoderm. *Cell and Tissue Research*.

[3] Kelley M. W. (2006). Regulation of cell fate in the sensory epithelia of the inner ear. *Nature Reviews Neuroscience*.

[4] Mulvaney J., Dabdoub A. (2012). Atoh 1, an essential transcription factor in neurogenesis and intestinal and inner ear development: function, regulation, and context dependency. *Journal of the Association for Research in Otolaryngology*.

[5] Basch M. L., Brown R. M., Jen H.-I., Groves A. K. (2016). Where hearing starts: the development of the mammalian cochlea. *Journal of Anatomy*.

[6] Cai T., Seymour M. L., Zhang H., Pereira F. A., Groves A. K. (2013). Conditional deletion of Atoh1 reveals distinct critical periods for survival and function of hair cells in the organ of Corti. *The Journal of Neuroscience*.

[7] Chen P., Johnson J. E., Zoghbi H. Y., Segil N. (2002). The role of Math 1 in inner ear development: uncoupling the establishment of the sensory primordium from hair cell fate determination. *Development*.

[8] Lavigne-Rebillard M., Pujol R. (1990). Auditory hair cells in human fetuses: synaptogenesis and ciliogenesis. *Journal of Electron Microscopy Technique*.

[9] Nayagam B. A., Muniak M. A., Ryugo D. K. (2011). The spiral ganglion: connecting the peripheral and central auditory systems. *Hearing Research*.

[10] Lavrik I. N. (2014). Systems biology of death receptor networks: live and let die. *Cell Death & Disease*.

[11] Li H., Song Y., He Z. (2018). Meclofenamic acid reduces reactive oxygen species accumulation and apoptosis, inhibits excessive autophagy, and protects hair cell-like HEI-OC1 cells from cisplatin-induced damage. *Frontiers in Cellular Neuroscience*.

[12] He Z.-H., Zou S.-Y., Li M. (2020). The nuclear transcription factor FoxG1 affects the sensitivity of mimetic aging hair cells to inflammation by regulating autophagy pathways. *Redox Biology*.

[13] Verzella D., Pescatore A., Capece D. (2020). Life, death, and autophagy in cancer: NF-*κ*B turns up everywhere. *Cell Death & Disease*.

[14] He Z., Guo L., Shu Y. (2017). Autophagy protects auditory hair cells against neomycin-induced damage. *Autophagy*.

[15] Furness D. N. (2015). Molecular basis of hair cell loss. *Cell and Tissue Research*.

[16] Jansson L., Kim G. S., Cheng A. G. (2015). Making sense of Wnt signaling—linking hair cell regeneration to development. *Frontiers in Cellular Neuroscience*.

[17] He Y., Tang D., Cai C., Chai R., Li H. (2016). LSD1 is required for hair cell regeneration in Zebrafish. *Molecular Neurobiology*.

[18] Croce C. M., Calin G. A. (2005). miRNAs, cancer, and stem cell division. *Cell*.

[19] Riccardi S., Bergling S., Sigoillot F. (2016). MiR-210 promotes sensory hair cell formation in the organ of corti. *BMC Genomics*.

[20] Ebeid M., Sripal P., Pecka J., Beisel K. W., Kwan K., Soukup G. A. (2017). Transcriptome-wide comparison of the impact of Atoh1 and miR-183 family on pluripotent stem cells and multipotent otic progenitor cells. *PLoS One*.

[21] Lu X., Shu Y., Tang M., Li H. (2016). Mammalian cochlear hair cell regeneration and ribbon synapse reformation. *Neural Plasticity*.

[22] Samarajeewa A., Jacques B. E., Dabdoub A. (2019). Therapeutic potential of Wnt and Notch signaling and epigenetic regulation in mammalian sensory hair cell regeneration. *Molecular Therapy*.

[23] Bai H., Jiang L., Wang X. (2019). Transcriptomic analysis of mouse cochleae suffering from gentamicin damage reveals the signalling pathways involved in hair cell regeneration. *Scientific Reports*.

[24] Waqas M., Zhang S., He Z., Tang M., Chai R. (2016). Role of Wnt and Notch signaling in regulating hair cell regeneration in the cochlea. *Frontiers of Medicine*.

[25] Savary E., Sabourin J. C., Santo J. (2008). Cochlear stem/progenitor cells from a postnatal cochlea respond to Jagged1 and demonstrate that Notch signaling promotes sphere formation and sensory potential. *Mechanisms of Development*.

[26] Brown R., Groves A. K. (2020). Hear, hear for Notch: control of cell fates in the inner ear by Notch signaling. *Biomolecules*.

[27] Brooker R., Hozumi K., Lewis J. (2006). Notch ligands with contrasting functions: Jagged1 and Delta1 in the mouse inner ear. *Development*.

[28] Liu Z., Owen T., Fang J., Zuo J. (2012). Overactivation of Notch1 signaling induces ectopic hair cells in the mouse inner ear in an age-dependent manner. *PLoS One*.

[29] Mizutari K., Fujioka M., Hosoya M. (2013). Notch inhibition induces cochlear hair cell regeneration and recovery of hearing after acoustic trauma. *Neuron*.

[30] Jayasena C. S., Ohyama T., Segil N., Groves A. K. (2008). Notch signaling augments the canonical Wnt pathway to specify the size of the otic placode. *Development*.

[31] Jacques B. E., Puligilla C., Weichert R. M. (2012). A dual function for canonical Wnt/*β*-catenin signaling in the developing mammalian cochlea. *Development*.

[32] Gómez-Orte E., Sáenz-Narciso B., Moreno S., Cabello J. (2013). Multiple functions of the noncanonical Wnt pathway. *Trends in Genetics*.

[33] Li W., Wu J., Yang J. (2015). Notch inhibition induces mitotically generated hair cells in mammalian cochleae via activating the Wnt pathway. *Proceedings of the National Academy of Sciences*.

[34] Oh S.-H., Johnson R., Wu D. K. (1996). Differential expression of bone morphogenetic proteins in the developing vestibular and auditory sensory organs. *The Journal of Neuroscience*.

[35] Granjeiro J. M., Oliveira R. C., Bustos-Valenzuela J. C., Sogayar M. C., Taga R. (2005). Bone morphogenetic proteins: from structure to clinical use. *Brazilian Journal of Medical and Biological Research*.

[36] Zhao G.-Q. (2003). Consequences of knocking out BMP signaling in the mouse. *Genesis*.

[37] Blauwkamp M. N., Beyer L. A., Kabara L. (2007). The role of bone morphogenetic protein 4 in inner ear development and function. *Hearing Research*.

[38] Yang X., Castilla L. H., Xu X. (1999). Angiogenesis defects and mesenchymal apoptosis in mice lacking SMAD5. *Development*.

[39] Liu S., Li W., Chen Y., Lin Q., Wang Z., Li H. (2011). Mouse auditory organ development required bone morphogenetic protein signaling. *Neuroreport*.

[40] Klagsbrun M. (1989). The fibroblast growth factor family: structural and biological properties. *Progress in Growth Factor Research*.

[41] Ladher R. K., Wright T. J., Moon A. M., Mansour S. L., Schoenwolf G. C. (2005). FGF8 initiates inner ear induction in chick and mouse. *Genes & Development*.

[42] Schimmang T. (2007). Expression and functions of FGF ligands during early otic development. *The International Journal of Developmental Biology*.

[43] Hayashi T., Ray C. A., Bermingham-McDonogh O. (2008). Fgf20 is required for sensory epithelial specification in the developing cochlea. *The Journal of neuroscience : the official journal of the Society for Neuroscience*.

[44] Jacques B. E., Dabdoub A., Kelley M. W. (2012). Fgf signaling regulates development and transdifferentiation of hair cells and supporting cells in the basilar papilla. *Hearing Research*.

[45] Huh S.-H., Warchol M. E., Ornitz D. M. (2015). Cochlear progenitor number is controlled through mesenchymal FGF receptor signaling. *eLife*.

[46] Cobourne M. T., Xavier G. M., Depew M. (2009). Sonic hedgehog signalling inhibits palatogenesis and arrests tooth development in a mouse model of the nevoid basal cell carcinoma syndrome. *Developmental Biology*.

[47] Watson S., Serrate C., Vignot S. (2010). Sonic Hedgehog signaling pathway: from embryology to molecular targeted therapies. *Bulletin du Cancer*.

[48] Hu X., Huang J., Feng L., Fukudome S., Hamajima Y., Lin J. (2010). Sonic hedgehog (SHH) promotes the differentiation of mouse cochlear neural progenitors via the Math1-Brn3.1 signaling pathway in vitro. *Journal of Neuroscience Research*.

[49] Brown A. S., Epstein D. J. (2011). Otic ablation of smoothened reveals direct and indirect requirements for Hedgehog signaling in inner ear development. *Development (Cambridge, England)*.

[50] Waldman E. H., Castillo A., Collazo A. (2007). Ablation studies on the developing inner ear reveal a propensity for mirror duplications. *Developmental Dynamics*.

[51] Eshraghi A. A., Wang J., Adil E. (2007). Blocking c-Jun-N-terminal kinase signaling can prevent hearing loss induced by both electrode insertion trauma and neomycin ototoxicity. *Hearing Research*.

[52] Eshraghi A. A., He J., Mou C. H. (2006). D-JNKI-1 treatment prevents the progression of hearing loss in a model of cochlear implantation trauma. *Otology & Neurotology*.

[53] Staecker H., Jokovic G., Karpishchenko S. (2019). Efficacy and safety of AM-111 in the treatment of acute unilateral sudden deafness-a double-blind, randomized, placebo-controlled phase 3 study. *Otology & Neurotology*.

[54] Lorito G., Hatzopoulos S., Laurell G. (2011). Dose-dependent protection on cisplatin-induced ototoxicity - an electrophysiological study on the effect of three antioxidants in the Sprague-Dawley rat animal model. *Medical Science Monitor*.

[55] Villani V., Zucchella C., Cristalli G. (2016). Vitamin E neuroprotection against cisplatin ototoxicity: preliminary results from a randomized, placebo-controlled trial. *Head & Neck*.

[56] So H., Kim H., Kim Y. (2008). Evidence that cisplatin-induced auditory damage is attenuated by downregulation of pro-inflammatory cytokines via Nrf2/HO-1. *Journal of the Association for Research in Otolaryngology : JARO*.

[57] Liu Y., Okada T., Shimazaki K. (2008). Protection against aminoglycoside-induced ototoxicity by regulated AAV vector-mediated GDNF gene transfer into the cochlea. *Molecular Therapy*.

[58] Isgrig K., McDougald D. S., Zhu J., Wang H. J., Bennett J., Chien W. W. (2019). AAV2.7m8 is a powerful viral vector for inner ear gene therapy. *Nature Communications*.

[59] He C., Zheng S., Luo Y., Wang B. (2018). Exosome Theranostics: biology and translational medicine. *Theranostics*.

[60] Breglio A. M., May L. A., Barzik M. (2020). Exosomes mediate sensory hair cell protection in the inner ear. *The Journal of Clinical Investigation*.

[61] György B., Sage C., Indzhykulian A. A. (2017). Rescue of hearing by gene delivery to inner-ear hair cells using exosome-associated AAV. *Molecular Therapy*.

[62] Li G., Chen K., You D. (2019). Laminin-coated electrospun regenerated silk fibroin Mats promote neural progenitor cell proliferation, differentiation, and survival in vitro. *Frontiers in Bioengineering and Biotechnology*.

[63] Koehler K. R., Nie J., Longworth-Mills E. (2017). Generation of inner ear organoids containing functional hair cells from human pluripotent stem cells. *Nature Biotechnology*.

[64] Sun G., Liu W., Fan Z. (2016). The three-dimensional culture system with matrigel and neurotrophic factors preserves the structure and function of spiral ganglion neuron in vitro. *Neural Plasticity*.

[65] Kayyali M. N., Wooltorton J. R. A., Ramsey A. J. (2018). A novel nanoparticle delivery system for targeted therapy of noise-induced hearing loss. *Journal of Controlled Release*.

[66] Lee M. Y., Park Y.-H. (2018). Potential of gene and cell therapy for inner ear hair cells. *BioMed Research International*.

[67] Hyakumura T., McDougall S., Finch S., Needham K., Dottori M., Nayagam B. A. (2019). Organotypic cocultures of human pluripotent stem cell derived-neurons with mammalian inner ear hair cells and cochlear nucleus slices. *Stem Cells International*.

[68] Zhang Y., Guo L., Lu X. (2018). Characterization of Lgr6+ cells as an enriched population of hair cell progenitors compared to Lgr5+ cells for hair cell generation in the neonatal mouse cochlea. *Frontiers in Molecular Neuroscience*.

